# Effect of Exercise Intensity and Duration on Cardiac Troponin Release

**DOI:** 10.1161/CIRCULATIONAHA.119.041874

**Published:** 2019-12-30

**Authors:** Lucy Marshall, Kuan Ken Lee, Stacey D. Stewart, Adam Wild, Takeshi Fujisawa, Amy V. Ferry, Catherine L. Stables, Hannah Lithgow, Andrew R. Chapman, Atul Anand, Anoop S.V. Shah, Neeraj Dhaun, Fiona E. Strachan, Nicholas L. Mills, Mark D. Ross

**Affiliations:** 1 British Heart Foundation Centre for Cardiovascular Science (L.M., K.K.L., S.D.S., A.W., T.F., A.V.F., C.L.S., A.R.C., A.A., A.S.V.S., N.D., F.E.S., N.L.M.), University of Edinburgh, United Kingdom.; 2 Usher Institute of Population Health Sciences and Informatics (N.L.M.), University of Edinburgh, United Kingdom.; 3 School of Applied Science, Edinburgh Napier University, United Kingdom (H.L., M.D.R.).

**Keywords:** physical exercise, troponin

The latest generation of high-sensitivity cardiac troponin assays are able to quantify very low concentrations of troponin in the majority of people. Indeed, international guidelines now recommend the use of low concentrations of troponin to risk-stratify patients with suspected acute coronary syndrome.^1^ Furthermore, troponin is an independent predictor of cardiovascular events in apparently healthy populations, and therefore, concentrations within the reference range may have a wider role in guiding the use of preventative therapies.^2^ However, troponin can become significantly elevated after physical exercise. Interpreting troponin concentrations in this context is challenging because the kinetics of troponin release after physical exercise are not well understood. Most previous research has used assays that are not able to quantify troponin within the reference range, or has involved endurance athletes and evaluated a single time point rather than serial sampling.^3^

In this study, we evaluated the effect of intensity and duration of physical exercise on troponin concentration in 10 physically active healthy volunteers (7 men and 3 women; age, mean±SD, 34±7 y). The study was approved by the Research Ethics Committee and was conducted with written informed consent. Eligibility was assessed using the Physical Activity Readiness Questionnaire, the American Heart Association Pre-Participation Screening Questionnaire, and a 12-lead electrocardiogram. All participants underwent an initial fitness test using a maximal graded exercise test on a cycle ergometer to exhaustion to quantify their lactate threshold. They subsequently attended 3 study visits, at least 7 days apart, during which they underwent exercise on a cycle ergometer. The first visit involved low-intensity cycling (50%–60% of the participant’s lactate threshold) for 60 minutes. During the second visit, participants cycled at high intensity (80%–90% lactate threshold) for 60 minutes, and during the third study visit, participants cycled at moderate intensity (60%–70% lactate threshold) for 4 hours. Troponin concentrations were measured at the start of exercise and then hourly for up to 6 hours during each study visit, followed by measurements at 1, 2, and 7 days thereafter using the ARCHITECT_STAT_ high-sensitive troponin I assay (Abbott Laboratories). This assay has a limit of detection of 1.2 ng/L and an upper reference limit (99th percentile) of 34 ng/L in men and 16 ng/L in women. Change in troponin concentration within each study visit was evaluated by a 1-way repeated-measures ANOVA and paired *t* tests. We compared troponin concentrations between visits by a 2-way repeated-measures ANOVA.

Study participants had a median troponin concentration of 1.8 ng/L (interquartile range, 0.8–5.7) at baseline. Troponin was elevated after moderate- and high-intensity exercise (1-way ANOVA, *P*<0.001 for both), but was unchanged after low-intensity exercise (*P*=0.055). Troponin concentrations were significantly higher after the shorter duration of high-intensity exercise (peak, 13.0 ng/L [6.5–27.1]) compared with the longer duration of moderate-intensity exercise (6.9 ng/L [2.9–7.9]; 2-way ANOVA, *P*=0.028). After moderate- and high-intensity exercise, troponin concentration returned to baseline within 48 hours (Figure). The median heart rate and peak power output were 112 bpm (interquartile range, 99–142) and 113 W (88–135), respectively, during low-intensity exercise, 151 bpm (135–162) and 139 W (127–159) during moderate-intensity exercise, and 155 bpm (139–165) and 178 W (161–205) during high-intensity exercise.

**Figure.**
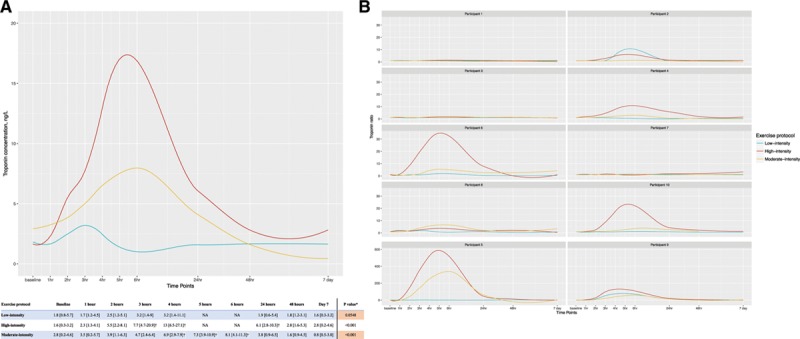
**The effect of exercise intensity and duration on cardiac troponin release. A**, Median high-sensitivity cardiac troponin I concentrations after low-, moderate-, and high-intensity exercise. Values are median (interquartile range) in ng/L; n = 10 participants. Statistical tests performed on log-transformed troponin ratio relative to baseline concentration at each time point. **B**, Individual participant time-activity curves illustrating fold-changes in cardiac troponin I concentration from baseline. *One-way repeated-measures ANOVA comparing troponin concentration across time points. †Significant difference from baseline concentration using paired t tests (*P* value <0.05). NA indicates not assessed.

Our findings suggest that the magnitude of troponin release is more significantly associated with intensity than duration of physical exercise. The underlying mechanisms of release after exercise are still incompletely understood. It is thought that a small pool of troponin exists unbound within the cytosol of cardiomyocytes. Mechanical shear stress as a result of the hemodynamic response to strenuous physical activity may transiently increase cell membrane permeability, leading to the release of troponin from this cytosolic pool. Differences in the cellular mechanisms of release are likely to explain why the duration of exercise-induced troponin release is significantly shorter than in acute myocardial infarction, where troponin concentrations remain elevated for >1 week.

We observed significant heterogeneity in the magnitude of troponin release across individual participants, with the ratio of peak troponin concentration compared with baseline ranging from 2 to 600 across individual participants. Although only 3 participants had troponin concentrations above the 99th percentile after moderate or high-intensity exercise, 9 of 10 had concentrations above the threshold of 5 ng/L used to risk-stratify patients with suspected acute coronary syndrome.^4^ This has important clinical implications given the increasing use of early rule-out pathways, which use low concentrations of troponin to make clinical decisions. Our data would suggest that the intensity, duration, and elapsed time since physical exercise should be considered when interpreting troponin concentrations. Furthermore, recent reports suggest that greater exercise-induced troponin release may be associated with higher risk of future adverse cardiovascular events.^5^ Further research is required to understand the determinants of heterogeneity in this response to exercise and the clinical significance of these exercise-induced troponin elevations, particularly in individuals with lower cardiorespiratory fitness.

## Acknowledgments

We thank Marina Mocognie, Russell Wilson, and Neil Guthrie, laboratory technicians at Napier University, for their assistance in the study.

## Sources of Funding

This study was funded by the British Heart Foundation through a Clinical Research Training Fellowship (FS/18/25/33454), a Senior Clinical Research Fellowship (FS/16/14/32023), and a Research Excellence Award (RE/18/5/34216).

## Disclosures

Drs Lee, Chapman, Anand, and Shah have received honoraria from Abbott Diagnostics. Dr Mills reports research grants awarded to the University of Edinburgh from Abbott Diagnostics and Siemens Diagnostics outside the submitted work, and honoraria from Abbott Diagnostics, Siemens Diagnostics, Roche Diagnostics and Singulex. The other authors report no conflicts.
